# Quality early childhood education and care in a time of COVID-19

**DOI:** 10.1177/1476718X231153105

**Published:** 2023-02-11

**Authors:** Sasha Tregenza, Verity Campbell-Barr

**Affiliations:** University of Plymouth, UK

**Keywords:** adaptability, COVID-19, early childhood education and care, free play, health and safety, parent partnership, quality

## Abstract

Contextual approaches to high quality Early Childhood Education and Care (ECEC) seek to capture the complexity of children’s lives, developing pedagogical approaches that are responsive to children’s needs and interests. In 2020, the COVID-19 pandemic provided a complex layer to the question of what constitutes quality ECEC. A mixed methods appreciative inquiry of educators’ and parents’ views of quality in one ECEC setting in England, became an unexpected ethnographic exploration of quality ECEC in the time of a global pandemic. The findings indicate how features of quality, such as offering a range of learning environments and structuring the pedagogic environment to offer free-flowing play, had to be adapted to prevent the spread of COVID-19. The focus on quality shifted, prioritising the health and safety of families and staff, over the quality and variety of the curriculum. Greater emphasis was also placed on children’s social and emotional well-being to support their ability to understand and manage the changes to routines in response to the pandemic. The findings demonstrate that the early years workforce remains central to understanding and supporting quality, concluding that quality ECEC is shaped by adaptability – adapting to the needs of children, families, staff, and the unprecedented context of COVID-19. The focus on adaptability seeks to highlight how educators frequently respond to unique contexts in juggling concepts of quality ECEC. Consequently, a recommendation is made for future educator training to consider the importance of adaptability, in providing a useful framework for reimaging quality ECEC post COVID-19.

## Introduction

In this article, we explore contextual responses to quality early childhood education and care (ECEC) at the time of the global COVID-19 pandemic. Quality ECEC has been critically presented as a social construct (Dahlberg et al., 2013), requiring contextual responses to the question of what is quality? (Moss et al., 2016). Proponents of contextual constructs of quality ECEC are frequently positioned as being in opposition to more universal models (Dahlberg et al., 2013), illustrating the challenges surrounding the identification of quality, owing to individual ontological and epistemological perspectives (Crotty, 1998). However, in 2020 there was a new context for the question of what quality ECEC is, due to COVID-19. The pandemic presented a new dynamic in how to provide contextual responses to the question of quality, with early years educators around the world reconsidering how they provide high quality pedagogical practice for the children they work with.

To explore quality during a time of uncertainty we draw on an Appreciative Inquiry (AI) of Sasha’s ECEC setting in England, that set out to consider interpretations of quality from the perspective of parents and educators. AI is a strengths-based approach to systems change, focussed on positive idea generation as a social and cultural process, explained further within the methods section. The fieldwork work coincided with the outbreak of COVID-19 (spring/summer 2020), resulting in an unexpected ethnographic study of quality in the time of a global pandemic, as aspects of quality ECEC evolved to meet the demands of a new context. Locating the research in the international literature on quality ECEC helps to illustrate how this study of one ECEC setting in England, represents the story of thousands of settings around the world, as educators provided quality ECEC during COVID-19. The findings illustrate how aspects of pedagogical practice once viewed as central to quality had to change, as did pedagogical environments and social interactions. Further, the relationships between parents, educators and the place of the ECEC setting in the wider social community also shifted. Despite challenges to concepts of quality ECEC as a response to COVID-19, the research also illustrates opportunities for positive developments during this period, such as strengthening staffing practices. Staffing practices during COVID-19 reflect the continued importance of educators for quality ECEC, drawing attention to their ability to adapt and respond to the context.

## Literature review

Quality ECEC continuously evolves causing ongoing debate surrounding its definition, often influenced by academic research and policies (Bonetti and Brown, 2018). Frequently characterised as process and structural quality (the former relating to the features that can be measured and regulated, the latter the daily experiences and routines), quality ECEC has been constructed as something ‘out there’ waiting to be discovered, whereby to achieve quality the individual components which enable success need to be defined (Dahlberg et al., 2013).

Fenech (2011) outlines the evolutions in research around quality ECEC, highlighting three ‘waves’ of empirical research. The first focused on the effects of maternal and non-maternal care on children’s development; the second acknowledged the importance of quality, seeking to examine what comprised quality; whilst the third considered factors beyond quality (e.g. the environment and family background) which impacts child development. The discussion illustrates the well-versed connections between quality ECEC and child development, which in the English context has seen quality ECEC generally accepted as the ability to promote children’s progress to reach a Good Level of Development (Department for Education, 2021). In turn, the measures of child development have become associated with children being ‘school ready’, whereby they achieve pre-determined outcomes deemed to provide the foundations for their later school success (Bradbury, 2014). We do not subscribe to a definition of quality that is determined solely by school readiness and identify that the closure of education provision during periods of the COVID-19 pandemic, offers opportunities to reconceptualise what determines understandings of quality.

Quality has been identified as a process of conforming to norms and something that can be achieved when working in a desired way (Moss, 2016). In England, quality (as driven by the inspecting body Ofsted) has been identified as a data driven exercise whereby educators document their conforming to the norms (Bradbury and Roberts-Holmes, 2017). Ofsted grade ECEC settings against their compliance to standards, with a grade of Outstanding, Good, Requires Improvement or Inadequate. However, data driven models of quality are seen to lack acknowledgement of the subtleties of ECEC practice, such as how an educator responds to the needs of the children in their care, or the nuances of the of the daily rhythms of an ECEC setting (Georgeson, 2018). Whilst numerous definitions of quality ECEC exist, this research values Smith et al.’s (2000) concept, as it provides a comprehensive and constructed explanation from a societal, educational, and parental viewpoint:

*The essential components of early childhood environments which are valued in our society, and which support the well-being, development and rights of children, and support effective family functioning.*

(Smith et al., 2000: 48)

Acknowledgement is given towards how this explanation does not address what these essential components are, but this seems apt in considering quality at a time when what is valued in society has shifted dramatically due to COVID-19.

Numerous studies have demonstrated a relationship between quality ECEC and staff qualifications (Callanan et al., 2017; Sylva et al., 2004) and anecdotally, those working in and alongside ECEC identify the importance of qualifications for quality (Georgeson and Payler, 2014). Evidence indicates the complex array of subject specific knowledge that is present within ECEC qualifications and the forming of knowledge combinations for informing professional practice. Educators often identify the inclusion of elements beyond the tangible knowledge studied as a part of a formal qualification. Acknowledging the role that attitudes and dispositions play in contributing to professional knowledge and practice, in nurturing and educating the children that they work with (Andrew, 2015; Brock, 2006).

Whist components of quality, such as professional knowledge, are regularly discussed, it is also important to consider different interpretations of quality ECEC from different positions (Urban, 2008). For example, Callanan et al. (2017) considered how parents define high-quality provision through word-of-mouth, adult to child ratio, good staff retention, qualified and experienced educators, resources, and safety. Despite evidence that parents can overestimate the quality of provision (Grammatikopoulos et al., 2014) and can lack an understanding of reports on quality (Mathers et al., 2012), identification is given towards how educators who respond to the needs and emotions of children and families are important in developing parent’s understandings of quality ECEC (Mathers et al., 2014). Furthermore, Callanan et al. (2017) identified three themes to ensure good practice: tailoring practice to meet the needs of children, having skilled and experienced educators and retaining an open and reflective culture, indicating the importance of valuing parents and educator’s views of quality ECEC to strengthen practice.

The interchangeable components of quality and interpretations of quality from different positions discussed thus far, link to Wood (2019) comparing ECEC to a kaleidoscope. The picture of ECEC and how it should look changes every time the kaleidoscope is turned, owing to developing research or understanding. In March 2020, the kaleidoscope took an unexpected turn, with pedagogical practice, professional knowledge and children’s needs all having a new light shined through them. COVID-19 is an infectious virus that has affected the world resulting in several measures to limit social interaction to minimise its spread. In England, this included national lockdowns that saw ECEC settings providing a service for just the children of essential workers and those identified as vulnerable. ECEC settings were asked to prioritise the care and safety of the children still attending and to ensure these children continued to benefit from a range of educational activities (Department for Education, 2021). However, the delivery of the curriculum had to alter to coincide with procedures to limit the spread of COVID-19 (Gov.uk, 2021).

The literature discussed indicates that there are various methods of identifying and interpreting quality ECEC. For this reason, the research adopted a multimethod, multi-perspective approach to the question of what constitutes quality ECEC. The research presented focuses on the period of the first national lockdown in England, but we acknowledge that there have been subsequent lockdowns, with varying social restrictions. At the time of writing there were global variations in whether ECEC remained open, highlighting the need to consider contextual responses to the question of quality ECEC during the pandemic.

## Methods

To further identify understandings of quality ECEC, this research set out to explore parental and educators’ interpretations of ‘outstanding’ practice, within an English setting located on the periphery of a town in the South West. The setting provides ECEC to children from birth to 5-year-old, consisting of five rooms: two rooms for the under-twos, one room for the 2-year-olds and two pre-school rooms (children aged 3 and 4). Staff are assigned to one room, but may move around during busy periods, depending on ratios and support needed (Table 1):

**Table 1.** A table to present the qualifications held by staff within the setting.

	Childcare room		
Educator level of qualification *L* = Level	Under twos area	2-Year-old room	Pre-school area
	Trainee L3	L3	Trainee L3
	L3	L3	L3
	L3	L3	L5
	L3	L3	L5
	L3		L5
			Early Years Teacher
			Early Years Teacher

This research process aimed to uncover an in-depth view of existing quality ECEC within the setting and enhance this further by uncovering the participants perceptions of quality. Therefore, the 4D model of Appreciative Inquiry (AI), developed by Cooperrider (1986) was adopted within this research. This 4D model focuses on the identification of what is working well, to discover understandings and use these discoveries to promote effectiveness and integrity within a social system, becoming an alternative to problem-solving (Shuayb et al., 2009). Where action-research has focused on identifying issues in need of development, AI identifies the good practice to embed this further within a setting. AI does not involve problem solving, but instead aims to discover, understand, and support innovations, combining social-organisational values with social-organisational practices. Through focussing on best practice, insight is generated as to what is possible and how to extend best practice. Thought was given to AI potentially creating an illusion that the extraordinary exists continuously, by solely focusing on positives (Cooperrider and Srivastva, 1987). This reflects Bright’s (2009) perception that improving something already working well, has less impact than identifying problematic practice. Suggesting how enhancing aspects of quality already in place needs to become part of the naturally functioning flow of everyday practice, similar to how the process of changing problematic practice has become the norm.

Various techniques for conducting an AI exist (Bushe, 2011), thus questioning which method is most successful in maintaining a lasting impact. The 4D model of AI used within this research is described as the almost universally accepted method, suggesting the delivery of successful outcomes (Cooperrider et al., 2008). The 4D model is a four-part investigation: ‘discovery’, where participants reflect upon what is working well; ‘dream’ sharing collective desires to establish a common goal; ‘design’ to collaboratively create a proposal and ‘destiny’ to determine how this will be achieved (Cooperrider et al., 2008). The ‘discovery’ and ‘dream’ sections were achieved via a questionnaire, to identify perceptions of quality ECEC from parents and educators. The questionnaires were shaped by the literature alongside Ofsted guidance. Themes emerging from the questionnaire data (as discussed in the approach to analysis) shaped a focus group with educators. The focus group supported the ‘design’ and ‘destiny’ to enhance existing quality within the setting. Biesta (2017) describes how this mixed-method approach is influential in developing understandings within social sciences, strengthening the findings through uniting responses from the questionnaires and focus group, to triangulate outcomes and create innovative concepts of quality ECEC. Whilst mixed-method approaches generate complex data analysis, the opportunities produced by combining methods outweighed the challenge of analysis (Cohen et al., 2011).

The following procedure outlines the research process:

- Ethical approval obtained.
- Consent gained from Chief Executive Officer of the setting involved.
- Opportunity to participate offered to all parents and educators involved with the setting.
- A pilot educator and a pilot parent questionnaire completed.
- Questionnaires distributed to 16 consenting educators, all questionnaires completed and returned.
- Questionnaires distributed to 32 consenting parents, of which 12 questionnaires completed and returned.
- Responses from the questionnaires analysed, developing discussion points for the focus group. Focus group offered to all educators, of which seven of the 16 participated. The focus group took place on the 23rd June 2020.

### An unexpected ethnography

During the research, the COVID-19 Pandemic occurred (World Health Organisation, 2020) creating implications for the research process and directly impacting practice, whereby expectations and understandings of quality ECEC shifted. COVID-19 created a situation where the perceptions of quality ECEC collected via the questionnaire responses pre-COVID, did not reflect the priorities of quality established throughout the pandemic. This resulted in an unexpected ethnographic method to emerge. As a teacher within the setting Shasha was part of this small-group context, sharing experiences with the participants, gaining an in-depth representation and multiple perspectives around the challenge of adapting pedagogy.

An ethnographic approach enabled theories around quality ECEC to emerge within a natural setting, providing insider accounts of the development of provision resulting from a change in social situation (Dennis and Huf, 2020). This formed a constructed view of quality ECEC throughout the pandemic. Consideration was given to this in-depth view existing within a specific small-group context causing limitations around generalisability, as the insider accounts of provision relate to this setting alone. Recognition is given to the inability to construct one single picture of the world and that this subjective and interpretive reflection on practice can only be acknowledged as a single definition of quality ECEC during COVID-19. However, other ECEC settings will relate to the identified views of quality ECEC encountered during the pandemic and the influence it has had on provision (Cohen et al., 2011).

The ethnographic approach identifies Sasha as both teacher and researcher (with Verity as advisor). From the outset it was important the participants understood that their responses were to be analysed, for the sole purpose of this research investigation without judgement relating to their professional knowledge (Cohen et al., 2011). This was discussed whilst gaining informed consent and managed by applying Brookfield’s (1995) Lenses Theory whilst constructing questionnaires and throughout the analysis. The process aimed to limit the level of influence Tregenza’s insider knowledge had upon the interpretation of responses, by ‘looking’ through the lens of the parents and educators, as opposed to a ‘teacher’s’ perspective. Responses were handled sensitively, with ethical integrity, to reassure participants of their anonymity (British Educational Research Association [BERA], 2018).

Ethical tensions can occur at any point, so procedures were required to manage concerns appropriately. One educator became apprehensive about taking part after providing consent. Despite ethical reassurances, the educator remained uncertain. Consequently, the educator chose to withdraw, and this was accepted without challenge (Hammersley, 2017). The ethical procedures ensured that the educator’s uncertainty was handled respectfully and professionally, enabling an informed judgement surrounding participation (BERA, 2018).

### Approach to analysis

The findings draw on aspects of the AI, whilst focusing on the changes to practice that were observed through the ethnography. Using rating scales, the questionnaire started to identify common perceptions of quality ECEC prior to COVID-19. Table 2 presents the most frequent responses to existing aspects of high-quality ECEC within the setting, shared from both the parents and educator’s perspectives.

**Table 2.** Summary of key results.

Topic	Number of responses			
	Strongly agree		Agree	
	Parents	Educators	Parents	Educators
The setting maintains the safety of children and the environment is secure	6/12	14/16	6/12	1/16
The experiences offered to children over time support development of the knowledge and skills needed for future learning	10/12	10/16	2/12	5/16
Educators show a firm understanding of early years learning and development and know how to promote this effectively	9/12	11/16	2/12	4/16

The most common shared aspects of quality ECEC within the setting, identified through the questionnaire responses pre-COVID-19 (Table 2), formed themes for the ‘discovery’ and ‘dream’ stage of the AI. Appreciating and recognising aspects of quality ECEC which already exist, to consider how these elements of quality could be further enhanced within the setting. However, analysis of the open responses identified additional themes that required further exploration as to their meaning and/or the lack of consensus as to their contribution to quality ECEC. Table 3 presents the most common open responses which generated themes to take forward within the focus group with educators for further discussion.

**Table 3.** Themes for further analysis.

Question	Respondents	Number of responses	Responses linked to:
Is there anything you feel we as a setting could do further to provide your child with the best start in life?	Parents	1/12	Providing a range of outdoor learning experiences
Is there anything you feel we could develop further?	Educators	4/16	Providing a range of outdoor learning experiences
How could we ensure these personal examples of high-quality learning experiences continue to occur more regularly?	Educators	6/16	Answers relating to consistent staffing
Are there any areas you believe we go above and beyond in?	Educators	8/16	Answers relating to parent partnerships

The discussion of the data therefore incorporates a consideration of the questionnaire responses, focus group data and ethnographic reflections during the lived experience of providing quality ECEC during COVID-19. The discussion is focused upon aspects of quality ECEC identified in tables two and three. The participant quotes included throughout the discussion were selected due to the detail given providing illustrative examples of the responses, relating to the most common themes of quality ECEC identified above.

## Discussion

### Providing a range of learning experiences

Relating back to the questionnaire responses previously identified, daily practice under ‘normal’ circumstances would focus on delivering quality provision within a range of environments, tuning into children’s interests and needs, observing interactions and making judgements in support of their development. Questionnaire respondents identified the importance of learning within a range of contexts:

Parents responses:

‘Education delivered in a range of environments – indoors, outdoors, actually going out into the real world and experiencing the reality of different environments’

‘New activities within a safe and secure environment’

‘A comfortable and safe environment that would allow my child to learn and explore’

Educator response:

‘The setting goes above and beyond in providing opportunities for different experiences and in treating all children equally’

However, the restrictions and impact of the first national lockdown in England consequently led to a shift in inclusive provision and the nature of the learning environment. Two groups formed: 1, those children with essential worker parents or carers and those identified as vulnerable attended the setting; 2, those who remained at home. The children who did attend the setting through lockdown, experienced a shift in the learning environment. ECEC became less focused on the delivery of high-quality education and more about high-quality care, ensuring the children were protected as much as possible from the risk of contracting COVID-19. COVID-19 risk assessments and health and safety procedures became the main components in delivering quality ECEC, which was important in reassuring parents that the setting was as safe as possible. The setting endeavoured to keep in touch with all families and encouraged home learning opportunities, providing weekly activities and interactive stories online, as an option for families to engage with. For children at home, opportunities promoted through physically attending a setting such as developing friendships and social interaction could not be provided virtually, due to the need to place health and safety as the dominant indicator of quality ECEC.

Post lockdown, the setting reopened to all children. COVID-19 continued to influence practice, with smaller class sizes and ‘bubbles’ (children remaining in the same small groups), removing all soft furnishings and limiting the number of resources accessible to the children. The questionnaire responses pre-COVID identified how safety is an existing determiner of quality within the setting (Table 2). However, throughout the pandemic health and safety constrained the learning experience due to limiting explorations of the environment and access to resources.

Part of the setting’s ethos in the delivery of high-quality provision consists of supporting each child’s independence, tuning into their interests, and facilitating child-initiated play. This aims to promote independence and choice within the environment, providing children with the autonomy to choose where to learn, what to explore and how this will be achieved. However, the educator discussion identified that whilst free flow would extend the quality of experiences and learning opportunities, the implementation of ‘free flow’ was inhibited due to COVID-19. The flow of access between the rooms had to be restricted to limit interactions between bubbles. Further, the children had designated times to go inside and outside, imposing a temporal and spatial restriction on the approach to quality practice.

The impact of COVID-19 required the resources accessible to the children to be limited, with hygiene procedures embedded. The impact of taking away soft furnishings meant that opportunities to wear role play outfits were reduced and whilst cosy quiet areas remained, these now consisted of wipe clean mats instead of cushions and teddy bears. Resources continued to remain minimised, but the setting aimed to reduce the impact of these changes, for example by encouraging children to make props for their role play instead.

The enhanced focus on health and safety in preventing the spread of COVID-19 required the setting to omit shared sensory experiences from the provision. This has had an impact on the quality of experiences that could be provided to the children, due to the loss of the sand tray, water tray, and tuff spot. Such activities usually provide engaging sensory provision, igniting limitless opportunities for interaction between children, sharing thoughts, ideas, questions and creating motivation to experiment and explore. Instead, the children were provided with their own individual bags of playdough, personal trays of sand and water to try and overcome this restriction, but the group interaction did not appear to be so effective. It also became apparent that trying to keep individual resources personal to each child is challenging, therefore this had to be monitored by an educator.

Additional time was also allocated for wiping the handles of the bikes (for example) in between each child’s use and wiping the resources before another bubble accessed the outdoor provision. Furthermore, the children and educators were required to wash their hands after their time outside. This has now become part of the daily routine and the children follow this without question, which is great for hygiene, but the notion of ‘flow’ has changed dramatically within the setting. While the flow in activities is an accepted challenge, there are questions as to what impact this will have on children’s learning. The hand washing and cleaning could potentially become a further limitation in future free-flow exploration, due to a heightened sense of fear of picking up germs or getting dirty.

A parental response to the questionnaire suggested that quality ECEC requires ‘education delivered in a range of environments’ (Table 3). Pre-COVID-19 the children were provided with regular beach and Forest school opportunities, along with trips to sing at a local residential home and Christmas performances at a local donkey sanctuary. These quality learning experiences had to be put on hold because of the pandemic, impacting the ability for children to experience the real world.

A discussion amongst educators throughout the focus group considered ways to further develop the quality of the child-led approach to learning and extend the range of learning experiences and opportunities for children during and post-COVID regulations. Thought was given to achieving this by extending the amount of time children have access to daily free flow provision, to deepen the level of engagement and enhance learning and development opportunities.

### Responding to children’s development

An impact upon children’s learning and development was noted upon returning to the setting post lockdown, as an academic-terms provision had been lost. The gap in attendance by children (of non-essential workers and vulnerable groups) prompted an informal ‘baseline’ to assess where children were within their learning. This provided evidence of a need to re-settle the children into the setting, due to the impact of lockdown requiring an enhanced focus on Personal Social Emotional Development (PSED). Whilst PSED underpins all other aspects of learning under normal circumstance, it became even more important to promote PSED, to support children’s well-being and transition into post-lockdown life. Interestingly, an educator noted pre-COVID-19, how quality ECEC requires a focus on ‘children’s emotional and social well-being as one of the main points’. Indicating, that whilst daily practice previously underpinned PSED, the shift in quality ECEC throughout COVID-19, brought PSED to the fore. A focus was placed on activities that support a range of feelings and emotions, whilst instilling the implementation of new hygiene routines. Ensuring that the children and parents felt confident and reassured that the setting was a safe place for children to explore.

The questionnaire identified how most parents considered care and education as being of equal importance (9/12 responses), but the focus group illustrated the need to adjust understandings of what constituted quality ECEC throughout COVID-19. Both the emphasis on care and the restrictions placed on learning opportunities were accepted by educators with comments such as ‘if Ofsted walked in, not like now, not during COVID-19, but before and after it has to be free flow. . .’ The response implies that the setting’s COVID-19 quality procedures have become embedded, with the new way of working being to prioritise the care of children and educators over Ofsted preferences or creating desirable learning environments. Whilst the questionnaires and focus group raised elements of practice that could be further built upon, such as time for more free flow provision, it became apparent that creating optimal learning environments were not a core priority. Quality ECEC became focused on adapting to and making the best out of the current situation, whilst working in partnership to create plans for future practice, once deemed safe to implement.

### Working in partnership with parents

The educator questionnaire pre-COVID-19 identified how educators felt the setting went above and beyond in parent partnerships with responses such as:

‘Our relationships with parents and carers including feedback and meeting children’s individual needs’.

‘Our bonds with parents’

During the delivery of quality ECEC under ‘usual’ circumstance, the educators invested time into communicating with parents, giving feedback and listening to what the parents and children needed, thus illustrating a genuine care and consideration for the children’s families. Pre-COVID the setting welcomed parents through the door to drop off and pick up their children. This helped to develop relationships with the parents, enabling them to see their children playing and their creations on display etc. To some extent, entering the setting enabled parents to observe part of the nursery day. In addition, the setting would usually hold stay and play events, to further develop relationships with parents. However, the COVID-19 restrictions changed this quality interaction, requiring parents to drop off and pick up at the front door. Whilst some parents fed back that this made daily transitions easier, it meant they no longer physically observed a snapshot of provision. Post-lockdown the educators still delivered feedback at the entrance, but were required to wear visors and socially distance, keeping 2 m away from the parents, creating a slight barrier in terms of working in partnership with families.

The educators focus on relationships with parents has required a re-think. For example, whilst parents were previously welcomed into the setting for parents’ evenings; the COVID-19 restrictions have meant that alternative methods of communication have had to be found. The implementation of the FAMLY app enabled parent evenings to be achieved virtually, through video calling. However, in instances when connections could not be made, appointments were conducted via telephone instead. Whilst important information regarding each child was shared with their parents, the restrictions in place created distance between the parents and educators. This highlighted that creating quality partnerships with parents is not just about effective communication but also requires other elements, such as enabling parents to become familiar with, and part of, the physical setting.

Interestingly, COVID-19 highlighted how it was not only the children that required an increased focus on emotional and social well-being, but families needed extra support from the educators too. Understandably some parents were uncertain around their children attending a setting during a pandemic. Therefore, the setting provided regular communications detailing the procedures in place, updates on recommended guidance and checked in regularly with families, ensuring that the open-door policy remained, albeit in a slightly different way. As parents trust that quality ECEC is provided, it was important to maintain this partnership and viewpoint.

### Consistency of staff

Despite the disruptions to partnerships with parents and the delivery of the curriculum resulting from COVID-19, the national lockdown also brought some positives. The educator questionnaire pre-COVID indicated how educators linked examples of their considered outstanding practice, to the importance of knowing each child and delivering effective provision to enable children to make good progress. Educators were asked how these high-quality examples of personal practice could occur more regularly (responses linked to Table 3):

‘Regular staff in each room’

‘Consistent staff’

‘Train all staff to do this, encourage all to know their key children and to plan for their next steps’.

The comments from staff implied consistent staffing facilitates learning, becoming aware of children’s needs and interests in support of their development. The questionnaires identified how educators felt that the busyness of the setting pre-COVID required educators to move around the different rooms as and when needed to meet ratios, which impacted the quality of practice delivered. The need for educators to stay in their bubbles enabled the positive effect that consistent staffing has upon daily practice to be observed. This further developed educator relationships, teamwork and children’s attachments to their key workers.

During the first lockdown, as less children were attending and consistent staffing was in place, the educators were not caught up in the busy flow of daily practice, enabling them to take a step back and view the provision through the eyes of the child. This helped ensure the resources were easily accessible and that the learning environment was inviting, freshened up and organised, despite the earlier identified challenges. The outdoor area was renovated to include a new decked area, a revamped mud kitchen, and resources were updated and organised. Improvements on this scale would not have been possible under ‘normal’ circumstances. Furthermore, the educators’ understanding of the importance of supporting PSED throughout COVID-19, brought a fresh perspective to observations as staff evaluated the resources that could be provided and how they would support children’s explorations in a safe way.

With the smaller group sizes, many educators were furloughed during the first lockdown period. The furloughed educators came back to work with a reignited passion for working within ECEC and the vital role they have in supporting children’s well-being and learning. Reflection has led to the belief that the COVID-19 pandemic has strengthened the quality of ECEC, as the positive mind-set and teamwork shared by educators, accomplished the challenge of delivering the adaptions to quality ECEC discussed.

## Conclusion

The purpose of this research was to identify parents and educators’ perceptions of quality ECEC. The research explored contextual approaches to quality, acknowledging the juggling act required of educators within ECEC settings, to meet developing facets of quality and the expectations of different stakeholders (Urban, 2008). Such facets include meeting children’s needs, having skilled educators and an enabling environment (Callanan et al., 2017). During COVID-19 the expectations of these facets shifted, in a time of uncertainty quality care took precedence over quality education. When faced with an unexpected world-wide pandemic, quality became less focused on ‘desirable’ elements of ECEC, instead valuing the ability to instantly adjust practice to meet the safety requirements of unprecedented situations. Quality ECEC is not simply delivering desirable practice to enable components of success to be met (Dahlberg et al., 2013), but is arguably the ability to adjust, transition, support and meet the needs of children, families, and educators, within any given situation. The connection between quality ECEC and the professional knowledge and skills of the workforce are well versed. COVID-19 has placed demands on the workforce to rethink their well-rehearsed applications and delivery of practice. Suggesting quality ECEC requires a recontextualisation of professional knowledge and skills, to reflect the adaptability required to meet the ever-changing components of quality (Wood, 2019). COVID-19 has demonstrated that what constitutes high-quality ECEC practice is not always certain nor predictable.

The adaptability of the workforce has parallels with the global adaptations to life, seen as a result of COVID-19, while bringing to the fore that children have also had to adapt. Both contextual responses to the question of quality (Dahlberg et al., 2013) and the ecological facets that lie beyond the ECEC setting in shaping quality (Dalli et al., 2012; Fenech, 2011), provide rejuvenated theoretical emphasis for models of quality that are not about predictable outcomes or measures. The constant shift and change towards understandings of quality ECEC, have long implied that determiners of quality are open to debate and adapt relating to changing needs and requirements (Bonetti and Brown, 2018). This research focused upon educator and parent’s interpretations of quality ECEC, concludes that regardless of situations faced, trusting and genuine relationships between educators, children and families, provide the unity needed to adapt and deliver quality ECEC.

### Recommendations for quality ECEC

This study of one setting in South West England demonstrates how the ECEC sector is able to adapt and respond to the needs of the children in their care, and, the nuances of the rhythm of ECEC provision irrespective of circumstance. However, wider evidence is indicating that the workforce is in a vulnerable position owing to the economic pressures of the pandemic and an uncertainty towards identifying future ‘normal’ provision. This uncertainty poses potential compromises to the prospect of quality ECEC (Bonetti et al., 2021). Professional adaptability cannot be considered an endless resource for securing quality ECEC, nor can adaptability be conflated with cuts to resources. The outcome of this research is to recognise adaptability as providing a useful framework for reimaging quality ECEC post COVID-19. Consideration should be given to educator training, supporting and promoting the skills needed to remain open-minded and adaptive within practice. A further recommendation requires policies and regulations to remain mindful that components of quality ECEC are not universal, permanent, or pre-determined. Standardised models of quality ECEC cannot account for unexpected situations. Therefore, there is scope to question whether individual settings could establish their own expectations of quality ECEC, in response to their unique contexts. Now, more than ever, is a time for moving away from predetermined standards and measurable aspects of quality, that presume a linear relationship to predetermined outcomes. We live in a world that cannot be guaranteed, nor normality taken for granted. The example of one setting in the South West of England has illustrated that ECEC provision can respond to uncertainty and that adaptability can underpin concepts of quality.
